# Supplementary material for: Learning curve and surgical outcome of robotic assisted colorectal surgery with ERAS program
Source: Sci Rep. 2022 Nov 29;12:20566. doi: 10.1038/s41598-022-24665-w (PMC9709162; doi:10.1038/s41598-022-24665-w)
Supplement: Supplementary file 2 — Supplementary Information 2. [file 41598_2022_24665_MOESM2_ESM.zip › RAL Raw data-Ñ[▒K/139/HS_ñw╝╨░O▒Kñσ.pdf]

正式摘要(電子簽章：000000234324838)

W83 / W83 ☐ 英文標題(注意:無英文姓名-請補病人基本檔!) ☐【讀取】憑證

臺中榮民總醫院

出院病歷摘要

轉入號： 轉出號：

[redacted]

(8)流水編號	
(9)入院日期	111/01/04 (2022) 直腸外科 W83 - 096
(10)轉科日期	
(11)出院日期	111/01/13 (2022) 住院天數計 9 日

診斷

- 入院 :1. Adenocarcinoma of retum at 4cm FAV s/p short course radiotherapy on 2021/12/20~24
  [Hx]
  1. Nonkeratinizing carcinoma of nasopharynx (left predominant), undifferentiated, with left neck LNs metastases, cT1N1M0, stage II, s/p IMRT 66Gy/33frs plus concurrent C/T with weekly CDDP 40mg/m2 x 5 weeks (20210615-20210803), clinical CR
  2. DM under OHA
- 出院 :1. Adenocarcinoma of retum at 4cm FAV posterior wall
  s/p short course radiotherapy on 2021/12/20~24
  s/p Robotic Low Anterior Resection Total Mesorectum Excision+ protective ilostomy on 2022/01/05
  ypT3N2a(5/21)M0, pMMR, p63-, CDX2+, PD, AI+, PI+, TuDp-, CRM-, DisMg 0.6cm-, TRG: 5
  stage IIIB
  [Hx]
  1. Nonkeratinizing carcinoma of nasopharynx (left predominant), undifferentiated, with left neck LNs metastases, cT1N1M0, stage II, s/p IMRT 66Gy/33frs plus concurrent C/T with weekly CDDP 40mg/m2 x 5 weeks (20210615-20210803), clinical CR
  2. DM under OHA

主訴

- constipation for 3m and bloody stool for 1m

病史

- This 49 years old female patient has a medical history of
  1. Nonkeratinizing carcinoma of nasopharynx (left predominant), undifferentiated, with left neck LNs metastases, cT1N1M0, stage II, s/p IMRT 66Gy/33frs plus concurrent C/T with weekly CDDP 40mg/m2 x 5 weeks (20210615-20210803), clinical CR
  2. DM under OHA
  According to her, she had constipation for 3m and bloody stool for 1m. No body weight loss was noted. Then, she went to our CRS OPD. At OPD, colonscopy was done and showed tumor at 4CM FAV. Biopsy was done and report was adenocarcinoma. During last hospitaliztion, tumor suervey was done. She had received short courseradiotherapy since 2021/12/20~24.
  This time, she was admitted for surgical intervention.

體檢發現

-
  - General appearance :
    - easy looking , conscious clear , without cardiopulmonary distress
    - Vital signs自動帶入date 體溫(BT) 脈搏(P) 呼吸(R) 血壓(BP) 身高(BH) 體重(BW) BMI
      20220104 36.1℃ 89次/分 18次/分 122/58mmHg 160.5cm 59.4kg 23.1
  - HEENT :
    - Eyes: no anemic conjunctiva, no icteric sclera, normal EOM
    - ENT: no otorrhea, no rhinorrhea, no erythematous mucosa, no injected tonsil, no deformity
  - Neck: supple, freely movable, trachea midline,no deformity, no engorged jugular vein , no palpable thyroid or lymph node enlargement
  - Chest:
    - Inspection: Grossly normal, symmetric and fully expansion, no accessory muscle used, no operation scar, no spider angiomata, no gynecomastia
    - Palpation: no palpable mass or nodule, no subcutaneous emphysema, no tenderness
    - Ausculation: clear breathing sound
  - Heart:
    - Percussion: normal heart size
    - Ausculation: regular heart beat, normal S1, S2, no gallop, no murmur
  - Abdomen:
    - Inspection: in
      - flat contour, symetrically, no scar, no stria, no dilated veins, no rashes
    - Ausculation: normoactive bowel sound, no abdominal bruits
    - palpation: soft, no muscle guarding or rigidity, no local tenderness, no rebounding pain, no palpable mass, no palpable pulsation
    - Percussion: no shifting dulllness, no hepatomegaly, no splenomagaly
  -

Peripheral pulsation :	CA	BA	RA	FA	PA	PTA	DPA
Right side :	++	++	++	++	++	++	++/+++
Left side :	++	++	++	++	++	++	++/+++

  - Genito-anus: no tenderness or palpable mass lesion or rectal shelf, normal external genitalia, normal anal tone, brown stool

CRS CONDITION

  - Colonoscope report: TUMOR AT 4CM FAV, S/P BIOPSY
  - CEA: 13.02 ng/ml
  - Imaging studies(Sono , CT of abdomen and chest Films):
    - 檢驗項目 :CT ABDOMEN-SURVEY 報告日期 :1101215
      主 訴：
      Result:Y Finding: Imaging Report Form for Colorectal Carcinoma (AJCC 8th) 1. Imaging Date: 20211210 Imaging Modality, Imaging by CT scan Imaging Protocol: CT of whole abdomen post IV and oral contrast enhancement 2. Tumor location / Size Location: Rectum Size: Measurable: 5.4 cm (largest diameter) 3. Tumor invasion Tumor invades non-peritonealized pericolonic or perirectal tissues 4. Regional nodal metastasis N1 | N1b Two or three regional lymph nodes are positive. 5. Distant metastasis (In this study) No or Equivocal 6. Other findings: 1) Segemtnal wall thickening about 5.4cm at rectum with perirectal fat stranding, in favor of rectal cancer. 2) Heterogenous poor enhancing lesion about 3.3cm in uterus, in favor of myoma. 3) Mild wall thickening at uterine cervix 4) UB wall thickening with contrast enhancement, R/I chronic cystitis. 5) No abnormal focal lesion in liver, GB, pancreas, bilateral kidneys and spleen. 6) A 1.0cm bone island at left pubic bone
      Impression: Rectal cancer
  -
    - Lung

手術日期及方法

- 手術日期及方法Robotic Low Anterior Resection Total Mesorectum Excision+ protective ilostomy on 2022/01/05

住院治療經過

- After admission, robotic assisted Low Anterior Resection with ileostomy was performed on 01/05. She drank water on 01/06, and then, ate liquid diet on 01/07. Now she can tolerated soft diet. Hemovac and CVC was removed on 01/11. We removed stitch on 01/12. Under stable condition, she was discharged on 01/13.

合併症

一般檢查

- 累積報告：

DATE	TIME	ALB	TP	BIL.T	BIL.D	ALKP	AST	ALT	LDH	AMY	LIPASE	NH3	Ferritin	UA
1110104	1713	4.1	6.8	0.29	0.08	62	15	13	132					
1110105	1500	3.6		0.40	0.09	62	17	15						
1110110	1206						13	5						

DATE	TIME	WBC	RBC	HGB	HCT	MCV	PLT	BLST	NEUT	BAND	SEG	LYM	MON	EOS	BAS
1110104	1709	1930	4.86	10.5	33.9	69.8	170				70.0	18.0	10.0	2.0	0.0
1110105	1501	3740	4.34	9.5	29.5	68.0	149		88.8			6.7	4.5	0.0	0.0
1110110	1210	3130		8.5	26.7		206		84.4			8.3	4.8	2.2	0.3

DATE	TIME	NA	K	CL	BUN	CREAT	eGFR	CA	Mg	P	CRP	ESR	PCT	D-Dimer	LACTATE
1110104	1713	142	4.2	104	15	0.77		9.5							
1110105	1500	141	3.5	104	12	0.76	85.97	8.4							
1110110	1206	139	3.7		9	0.71		8.6							

DATE	TIME	GLUCOSE	HbA1C	CHOL	TG	HDL	LDL	KETONE	CK	CKMB	TRO-T	PT-P	APTT-P	NT-pro BNP
1110104	1724		6.8											
1110104	2057											10.0	24.8	
1110105	1500	174												

特殊檢查

病理報告

- 活體組織切片病理報告　手術日期：**111/01/05**　病理號：**S1100651**

診斷醫師:陳志榮 報告日期:111/01/11
Pathologic diagnosis:
1. Rectum, robotic low anterior resection --- Adenocarcinoma, poorly differentiated. Tumor invaded to perirectal soft tissue, and metastasized to perirectal lymph nodes (5/21). The bilateral cut ends are free of tumor.
2. IMA root, dissection --- Unremarkable fibrovasculoadipose tissue. Negative for malignancy.
3. Rectum, cut end, distal, resection --- Negative for malignancy.
Ancillary study for diagnosis:
1. IHC stains for MLH1, MSH2, PMS2 and MSH6 are done on section B. All above markers are preserved.
2. P63 (-) and CDX2 (+) IHC stains done for section B.
Prognostic and predictive factor:
1. Tumor size: 3.5x3 cm.
2. Depth of invasion: Perirectal soft tissue.
3. Histologic grade: G3: Poorly differentiated.
4. Angiolymphatic invasion: Present.
5. Perineural invasion: Present.
6. Tumor deposits (discontinuous extramural extension): Not identified.
7. Macroscopic tumor perforation: Not identified.
8. Surgical Margins:
a) Proximal Margin: Uninvolved by invasive carcinoma, distance of tumor from margin: 12 cm.
b) Distal Margin: Uninvolved by invasive carcinoma, distance of tumor from margin: 0.6 cm.
c) Radial or Mesenteric Margin: Uninvolved by invasive carcinoma, distance of tumor from margin: 0.5 cm.
9. Lymph node status: Involved (5/21).
10. Extranodal involvement: Not identified.
11. Tumor regression grading S/P CCRT: No known presurgical therapy.
12. Type of polyp in which invasive carcinoma arose: Not identified.
13. Additional pathologic findings: Not identified.
14. Pathological TNM Stage: pT3N2a (According to the eighth edition, American Joint Committee on Cancer Staging Guidelines for Tumors).
15. TNM descriptors: Not applicable.
Gross description:
The specimen consists of 1) a segment of colon, 14.5 cm in length and 5 cm in proximal cut end circumference, 6 cm in distal circumference, and perirectal soft tissue. A gray tan firm annular elevated and centrally ulcerative tumor, 3.5x3 cm, is noted, 1 cm to the distal cut end. On cross section, the tumor invades to the mesocolic soft tissue with 10 mm to the radial margin. The both cut ends are free of tumor grossly. 2) IMA root, a piece of tan yellow soft tissue, 4x2x1 cm. 3) distal cut end, a piece of tan mucosa-covered soft tissue, 1.9x1x1 cm. Representative part for section: A) proximal cut end B) tumor with distal cut end C-E) tumor H-K) lymph nodes; All for section: F) specimen 2 G) specimen 3. Blue ink indicated serosa; yellow ink indicated radial margin.
Note:
1. The interpretation of MMR panel and recommended studies are as below: (1) No loss of nuclear expression of MMR proteins: low probability of microsatellite instability-high (MSI-H) . (2) Loss of nuclear expression of MLH1 and PMS2: testing for methylation of the MLH1 promoter and/or mutation of BRAF is indicated (the presence of a BRAF V600E mutation and/or MLH1 methylation suggests that the tumor is sporadic and germline evaluation is probably not indicated; absence of both MLH1 methylation and of BRAF V600E mutation suggests the possibility of Lynch syndrome, and sequencing and/or large deletion/duplication testing of germline MLH1 may be indicated). (3) Loss of nuclear expression of MSH2 and MSH6: high probability of Lynch syndrome (sequencing and/or large deletion/duplication testing of germline MSH2 may be indicated, and, if negative, sequencing and/or large deletion/duplication testing of germline MSH6 may be indicated). (4) Loss of nuclear expression of MSH6 only: high probability of Lynch syndrome (sequencing and/or large deletion/duplication testing of germline MSH6 may be indicated). (5) Loss of nuclear expression of PMS2 only: high probability of Lynch syndrome (sequencing and/or large deletion/duplication testing of germline PMS2 may be indicated). (6) Please note: There are exceptions to the above IHC interpretations. These results should not be considered in isolation, and clinical correlation with genetic counseling is recommended to assess the need for germline testing.
2. This case has been peer reviewed by two doctors.
Erratum: (2022/01/12)
Prognostic and predictive factor:
11. Tumor regression grading S/P SCRT: Absent, extensive residual cancer with no evident tumor regression (poor or no response, score 3).
#T-59600_2 #M-81403_2 3 1105 S0ER0F
#T-C4560_2 #M-81406_2 2 1105 000000

其它

出院時情況

- 3.改門診治療

出院指示

-

藥名	劑量	劑量單位	用法	頻次	數量	數量單位	現況	天數	單位
(口)Takepron OD tab 30mg	1	TAB	PO	QDAC	7	TAB	TH	7	天
Ultracet tab	1	TAB	PO	Q12H	14	TAB	TH	7	天
Voren-K tab 25mg	1	TAB	PO	BID	14	TAB	TH	7	天
Econ tab 40mg	1	TAB	PO	QID	28	TAB	TH	7	天
Vesicare tab 5mg	1	TAB	PO	QD	7	TAB	TH	7	天
DailyCare actibest tab	1	TAB	PO	QD	7	TAB	TH	7	天
CefaDROxil-500 cap	1	CAP	PO	Q12H	14	CAP	TH	7	天

出院計劃

主治醫師_2222H 林俊余_______________ / 住院醫師_2346C 陳怡豪_______________

正式摘要(電子簽章：000000234324838)

W83 / W83

doctorRank:B
